# 50 Years of Children’s HeartLink and the Partnerships in Brazil

**DOI:** 10.21470/1678-9741-2019-0426

**Published:** 2020

**Authors:** Ulisses Croti, Valdester Cavalcante Junior, Marcelo Biscegli Jatene, Andreas Tsakistos, Bistra Zheleva

**Affiliations:** 1 Hospital da Criança e Maternidade, São José do Rio Preto, SP, Brazil.; 2 Instituto do Coração da Criança e do Adolescente - Cirurgia Cardiovascular, Fortaleza, Ceará, Brazil.; 3 Pediatric Cardiac Surgery Unit - Instituto do Coração, Hospital das Clínicas, Faculdade de Medicina da Universidade de São Paulo, São Paulo, SP, Brazil.; 4 Children’s HeartLink, Minneapolis, United States.

On October 25th, 2019, Children’s HeartLink celebrated its 50th anniversary at its annual gala fundraising dinner. The organization was founded in 1969 by Dr. Joseph Kiser in Minneapolis, Minnesota, USA, to assist children with congenital heart disease^[1]^. The fundraiser was attended by over 600 guests, including donors, Children’s HeartLink medical volunteers, and representatives of Children’s HeartLink partner hospitals from Brazil, China, India, Malaysia, and Vietnam^[2]^. There was live streaming connecting the Gala Party in Minneapolis to various partner services around the world.

**Fig. 1:**
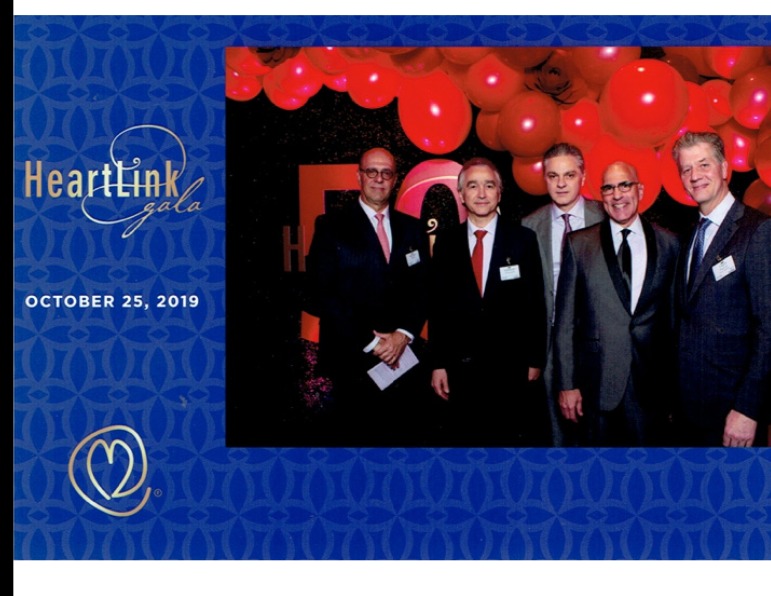
From right to left, Dr. David Overman, Dr. Joseph Dearani (both volunteers in Brazil), Dr. Ulisses Alexandre Croti, Dr. Valdester Cavalcante Pinto Junior and Dr. Marcelo Biscegli Jatene at the Children’s HeartLink Gala 2019.

Brazil was represented by three representatives of Children’s HeartLink partner hospitals: Dr. Ulisses Alexandre Croti, pediatric cardiac surgeon from Hospital de Base/Hospital da Criança e Maternidade in São José do Rio Preto, SP, partner since 2009; Dr. Valdester Cavalcante Pinto Junior, pediatric cardiac surgeon from Hospital de Messejana in Fortaleza, CE, partner since 2014; and Dr. Marcelo Biscegli Jatene, from Instituto do Coração - Hospital das Clínicas, Faculdade de Medicina da Universidade de São Paulo in São Paulo, SP, partner since 2019 (Figure 1).

The honoree of the evening was Dr. Joseph Dearani, Children’s HeartLink, who received the Children’s HeartLink Founders Award. Dr. Dearani is Chair of the Department of Cardiovascular Surgery at Mayo Clinic and Professor of Surgery in the Mayo College of Medicine. Dr. Dearani has been a Children’s HeartLink medical volunteer for 23 years and Medical Director of the Children’s HeartLink Board of Directors for 16 years. He has led Mayo Clinic medical volunteer teams on Children’s HeartLink training visits to Colombia, China, Brazil and India.

Throughout the evening, Children’s HeartLink President Jackie Boucher, United Healthcare Global Chief Executive Officer and presenting sponsor representative Molly Joseph, and Children’s HeartLink Board Chair Heather Hudnut Page shared with the guests what the organization has been able to achieve over its long history and serving more than a 1 million children over its 50 years. Children’s HeartLink’s current goal is to serve 1 million more children by 2030. In the words of Ms. Boucher, “We are celebrating our past and looking towards the future!”

**Fig. 2:**
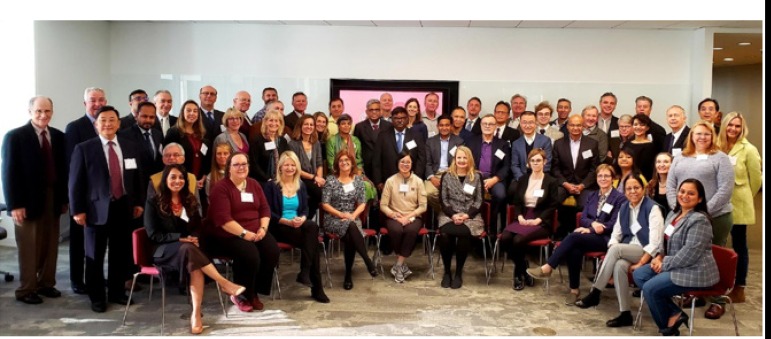
Group gathered during the first Children's HeartLink 50th Anniversary Summit 2019.

Continuing to celebrate the organization’s successes, Children’s HeartLink hosted on October 26^th^ the Children’s HeartLink 50th Anniversary Summit, a gathering of volunteer pediatric cardiac clinicians from around the word, partner hospital representatives, board members and International Advisory Board members (Figure 2). The goal of this summit was to outline possible future strategic directions and priorities for the global field of pediatric cardiac care, for Children’s HeartLink and the many partners with whom the organization works around the world. The summit discussions focused on four areas that Children’s HeartLink identified that need urgent attention globally: increasing capacity to care for children, training the medical workforce, closing the data gap, and financing. These were the four recommendations highlighted in The Invisible Child, a policy paper series on childhood heart disease and the global health agenda^[3,4]^ published in 2016. More specifically, the meeting had several objectives:

1. Discuss strategies to strengthen health systems and improve access to care for children and families affected by the onset of childhood heart disease by closing the data gap in congenital heart disease care, building the future pediatric cardiac workforce, building more capacity for pediatric cardiac care, and financing the scaling of pediatric cardiac care.
2. Define potential priorities that can serve as guidelines for nongovernmental organizations’ (NGOs) engagement in global cardiac care.
3. Identify opportunities for Children’s HeartLink to collaborate with other global NGOs, clinicians, academics, cardiac centers, policy makers, and patients and families.
4. Develop a global call to action for incorporating the needs of children with heart disease into existing global health strategies to increase sustainable and equitable pediatric cardiac care across the world.

The day ended with definitions of strategies to reach the goal of 1 million children with congenital heart disease treated with quality and sustainability in the next 10 years.

We, the representatives of Brazil, have experienced firsthand the benefits of this partnership between Children’s HeartLink and our hospitals, with significant improvements in the quality of care we provide to our patients, increase in our multidisciplinary team expertise, as well as in the number of children treated. We are encouraged to see the leadership and accountability with which Children’s HeartLink thinks about the future of pediatric cardiac services around the world, and we are even more confident and hopeful about new possible partnerships with other pediatric cardiac centers in our country^[5,6]^.
