# ERRATUM

**DOI:** 10.21470/1678-9741-2019-0426e

**Published:** 2020

In the editorial “50 Years of Children’s HeartLink and the Partnerships in Brazil”, with DOI code 10.21470/1678-9741-2019-0426, published in the Brazilian Journal of Cardiovascular Surgery, in edition 35.2, pages IV to VI, 2020:

In the original version the information was:

Ulisses Croti^1^, MD, PhD; Valdester Cavalcante Junior^2^, MD, PhD; Marcelo Biscegli Jatene^3^, MD, PhD; Andreas Tsakistos^4^, MD, PhD; Bistra Zheleva^4^, MD, PhD

The correct information is:

Ulisses Alexandre Croti^1^, MD, PhD; Valdester Cavalcante Pinto Junior^2^, MD, PhD; Marcelo Biscegli Jatene^3^, MD, PhD; Andreas Tsakistos^4^, MD, PhD; Bistra Zheleva^4^, MD, PhD

